# Regulation mechanisms of flavonoids biosynthesis of Hancheng Dahongpao peels (*Zanthoxylum bungeanum* Maxim) at different development stages by integrated metabolomics and transcriptomics analysis

**DOI:** 10.1186/s12870-022-03642-5

**Published:** 2022-05-21

**Authors:** Tao Zheng, Jun Han, Ke-xing Su, Bing-yin Sun, Shu-ming Liu

**Affiliations:** 1 Northwest Agriculture and Forestry University, College of Science, Yangling, 712100 China; 2 Forestry and Grassland Bureau of Xunhua Salar autonomous county, Xunhua, 811100 China; 3 Yangling Vocational &Technical College, Yangling, 712100 China

**Keywords:** *Zanthoxylum bungeanum* Maxim ‘Hancheng Dahongpao’, Transcriptome, Metabolome, Flavonoids biosynthesis

## Abstract

**Background:**

Flavonoids have strong free radical scavenging and antioxidant capacity. The high abundance of flavonoids in Chinese prickly ash peels have many benefits to human health. In this study, ‘Hancheng Dahongpao’, a main cultivar, was taken as materials to investigate the flavonoids biosynthesis mechanism of *Zanthoxylum bungeanum* Maxim at three key development stages by integration of metabolomics and transcriptomics analysis.

**Results:**

A total of 19 differentially accumulated metabolites were identified, the key flavonoids compounds were kaempferol, quercetin and their glycoside derivatives, and two major anthocyanins (peonidin O-hexoside and peonidin 3-O-glucoside). 5 gene networks/modules including 15 important candidate genes were identified, which was highly correlated with flavonoids. Among these genes, ZM-163828 and ZM-184209 were strongly correlated with kaempferol and quercetin, and ZM-125833 and ZM-97481 were controlled the anthocyanins biosynthesis. Moreover, it was shown that MYB-ZM1, MYB-ZM3, MYB-ZM5, MYB-ZM6 and MYB-ZM7 coordinately controlled flavonoids accumulation through regulating the structural genes.

**Conclusions:**

Generally, this study systematically revealed the flavonoids metabolic pathways and candidate genes involved in flavonoids biosynthesis and laid a foundation for the potential targets for the breeding of new valuable Chinese prickly ash cultivars.

**Supplementary Information:**

The online version contains supplementary material available at 10.1186/s12870-022-03642-5.

## Background

Flavonoids are a large class of secondary metabolites with strong biological activities generated in the process of long-term ecological adaptation of plants [1, 2]. Based on their structural differences, flavonoids are generally divided into nine main types, including chalcones, flavonoids, flavonols, dihydroflavonoids, flavanols, flavonoid glycosides, isoflavones, proanthocyanin and anthocyanins [3–5]. In plants, most flavonoids exist in the form of glycosides, and a few in the form of free state. The glycosylation sites are usually C-3 and C-7, and the glycosylation sites include glucose, raffinose, galactose, rhamnose, among which glucose is the most common. A variety of modifications such as glycosylation and methylation of flavonoids greatly enriched the derivatives varieties, and also improved the stability of these substances in plants [6, 7]. Flavonoids are widely distributed in flowers, fruits and leaves of many plants, about 4000 flavonoids have been found [8]. Flavonoids, with many types and complex structures, exhibit tissue-specificity and are regulated by genes and biotic and abiotic factors in environment [9].

Flavonoids in plants have enhanced plant resistance and chemical defense functions, and play an active role in plant ecological adaptation. Flavonoids play an important role in plant growth, development, flowering, fruit and antibacterial disease prevention. For example, flavonoids in roots can regulate root growth and affect mineral absorption, thereby affecting plant growth [10–12]. Flavonoids are usually related to plant stress resistance, which is beneficial to the absorption of reactive oxygen species (ROS) and prevent UV damage to plants [13, 14]. Flavonoids have many effects, such as anti-cardiovascular and cerebrovascular diseases, antioxidant, anti-aging, analgesic, anti-inflammatory, antitumor, antiviral and so on, which play an important role in human health. Numerous studies have confirmed that flavonoids have health functions, and further were processed into products with special functions such as health food and drugs [15].

The biosynthesis pathway of flavonoids has been investigated and identified from *Arabidopsis* and other plants [16]. The metabolic pathway of flavonoid biosynthesis is a complex process, include phenylalanine pathway, flavonoid biosynthesis and anthocyanins biosynthesis pathways [17]. Phenylalanine is catalyzed by phenylalanine enzyme (PAL), cinnamic acid 4-hydroxylase (C4H) and 4-coumarin coenzyme A ligase (4CL) to form coumarin coenzyme A, and the substrate further forms chalcone under the action of chalcone synthase (CHS) [18]. Naringin is generated by chalcone under the catalysis of chalcone isomerase (CHI). CHS and CHI are key enzymes in phenylalanine pathway [19]. Flavonoids were produced by naringenin catalyzed by flavanone 3-hydroxylase (F3H), flavonoids 3′-hydroxylase (F3’H), flavonoid 3′,5′-hydroxylase (F3’5’H) [20]. Dihydroflavonol reductase (DFR) is a key enzyme in the anthocyanin synthesis pathway, which catalyzes dihydromyricetin, dihydrokaempferol and dihydroquercetin to produce leucodelphinidin, leucopelargonidin and leucocyanidin, respectively. The above substances were catalyzed by anthocyanin synthase (ANS) to form unstable anthocyanin. Once formed, they will form stable anthocyanins through UDP-glycose/flavonoid glycosyltransferases (UFGT) [21]. There are mainly three types of transcription factors that regulate flavonoid biosynthesis in plants (MYB, bHLH and WD40), among which MYB are the predominant transcription factors [22]. Combined with metabolites and transcriptome analysis, the gene network and molecular regulation mechanism of flavonoid biosynthesis in many plants have been revealed, including *Arabidopsis* [23], *Ficus carica* Linn [16], tomatoes [24], tea plants [25], potatoes [26, 27] and *Ziziphus jujuba* Mill [22]. Therefore, the key gene networks controlling flavonoids biosynthesis in *Zanthoxylum bungeanum* Maxim peels during fruit development could be identified by integrating metabolites and gene expression profiles.

Chinese prickly ash (*Zanthoxylum bungeanum* Maxim) is a kind of plant resource with high edible and medicinal value, and has great economic and ecological value. In recent years, it was widely planted in China, and its planting area expanded rapidly [28, 29]. Its peel contains a variety of active ingredients with antibacterial, antitumor, antioxidant and other health effects, which are beneficial to human body [30, 31]. As the main polyphenol secondary metabolites in Chinese prickly ash plants, flavonoids have great benefits for many physiological activities of the human body [32]. However, the investigation on gene regulatory networks of flavonoids in Chinese prickly ash peels was lacking. In this study, the fresh fruits of Chinese prickly ash peels (*Zanthoxylum bungeanum* Maxim. ‘Hancheng Dahongpao’) at the three key development stages were picked as experimental materials, and the genes involved in flavonoids metabolism and the differential metabolite in Chinese prickly ash peels were systematically studied to reveal the gene networks related to flavonoids metabolism.

### Materials and Methods

#### Peel material

**Fig. 1:**
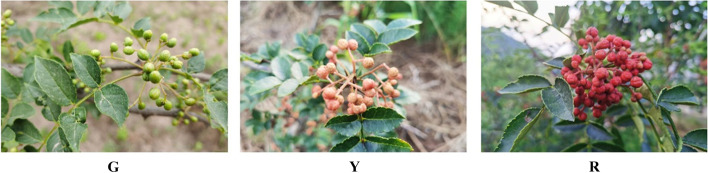
The fruits of ‘Hancheng Dahongpao’ at different development stages. G = green stage; Y = yellow stage; R = full red stage

The ‘Hancheng Dahongpao’ fruits at different development stages: green stage (G), yellow stage (Y) and full-red stage (R) (Fig. 1) were selected as test materials. The ‘Hancheng Dahongpao’ was planted in the experimental garden of college of forestry, Northwest A & F University, Yangling City (34°15’N, 108°03’E), Shaanxi Province, China. All peels uniformity in color without signs of mechanical damage or disease (approximately 2 mm) were washed with distilled water, frozen in liquid nitrogen and stored at −80 °C for further use. Three individual biological replicates (9 samples in total) were used for metabolism and transcriptome analysis of peels at each stage.

#### Sample preparation and metabolite extraction

All peels were treated by vacuum freeze-drying, grinded to powder (30 Hz, 1.5 min) by a mixer mill (MM 400, Retsch). The powder (100 mg) was dissolved in 1.0 mL extract (70% methanol aqueous solution), and the dissolved samples were vortexed (10 s, 40 Hz) every 10 minutes three times and kept at 4 °C overnight. Following centrifugation at 10, 000 g for 10 min, the supernatant was filtered by microporous membrane (0.22 μm pore size) and stored in the sampling bottle for UPLC-MS/MS analysis.

The mass spectrometry data were processed by the software analyst 1.6.3 software (AB SCIEX, Ontario, Canada). The partial least squares-discriminant analysis results (OPLS-DA) could maximize the distinction between groups and help to find differential metabolites. Based on the OPLS-DA results, the differential metabolites were screened by combining fold change and the variable importance in projection values (VIP). VIP ≥ 1 and fold change ≥2 or fold change ≤0.5 were set as the selection standard differential metabolites.

### RNA extraction, cDNA library construction and bioinformatics analyses

Total RNA extraction, detection and library construction from green, yellow and full-red peels were prepared by a previously described method. The cDNA libraries preparation was sequenced on the Illumina HiSeq 2000 platform, and 150 bp paired-end reads were generated. For gene-expression analysis, the clean reads were mapped to the reading of each gene by HTSeq v0.5.4p3 and the FPKM (fragments per kilobase of transcript per million mapped reads) method was used to estimate the reads count and expression levels of each gene, respectively.

#### Analysis of differentially expressed genes

Differentially expressed genes (DEGs) were identified based on the fold change of the FPKM values from the different samples and performed using DESeq2 v1.22.1. Following the difference analysis, the false discovery rate (FDR) control was applied to calculate the threshold of the P-value. The screening conditions for DEGs were |log2Fold Change| > = 1 and FDR < 0.05. The pathways of differentially expressed genes were annotated Based on the KEGG comprehensive database (Kyoto Encyclopedia of Genes and Genomes). WGCNA analysis (weighted gene coexpression network analysis) was carried out by WGCNA v1.69 to build a gene coexpression network based on genes coexpressed modules.

#### Experimental validation of candidate genes

Candidate genes expression analysis was performed with three independent biological replicates by using real-time quantitative PCR (RT-qPCR), which contained 10 differentially expressed structure genes and 3 MYB transcription factors genes. RT-qPCR analysis was performed on a Step One PULS real-time detection system detection system (ABI, Foster, CA, USA) using 2 × SG Fast qPCR Master Mix (High Rox, B639273, BBI, ABI). The candidate genes expression data was quantitatively calculated by 2 ^-ΔΔCT^ method with the reference gene UBQ. The primers and UBQ gene were shown in Table S1.

## Results

### Metabolome profiling in ‘Hancheng Dahongpao’ peels at different development stages

**Fig. 2:**
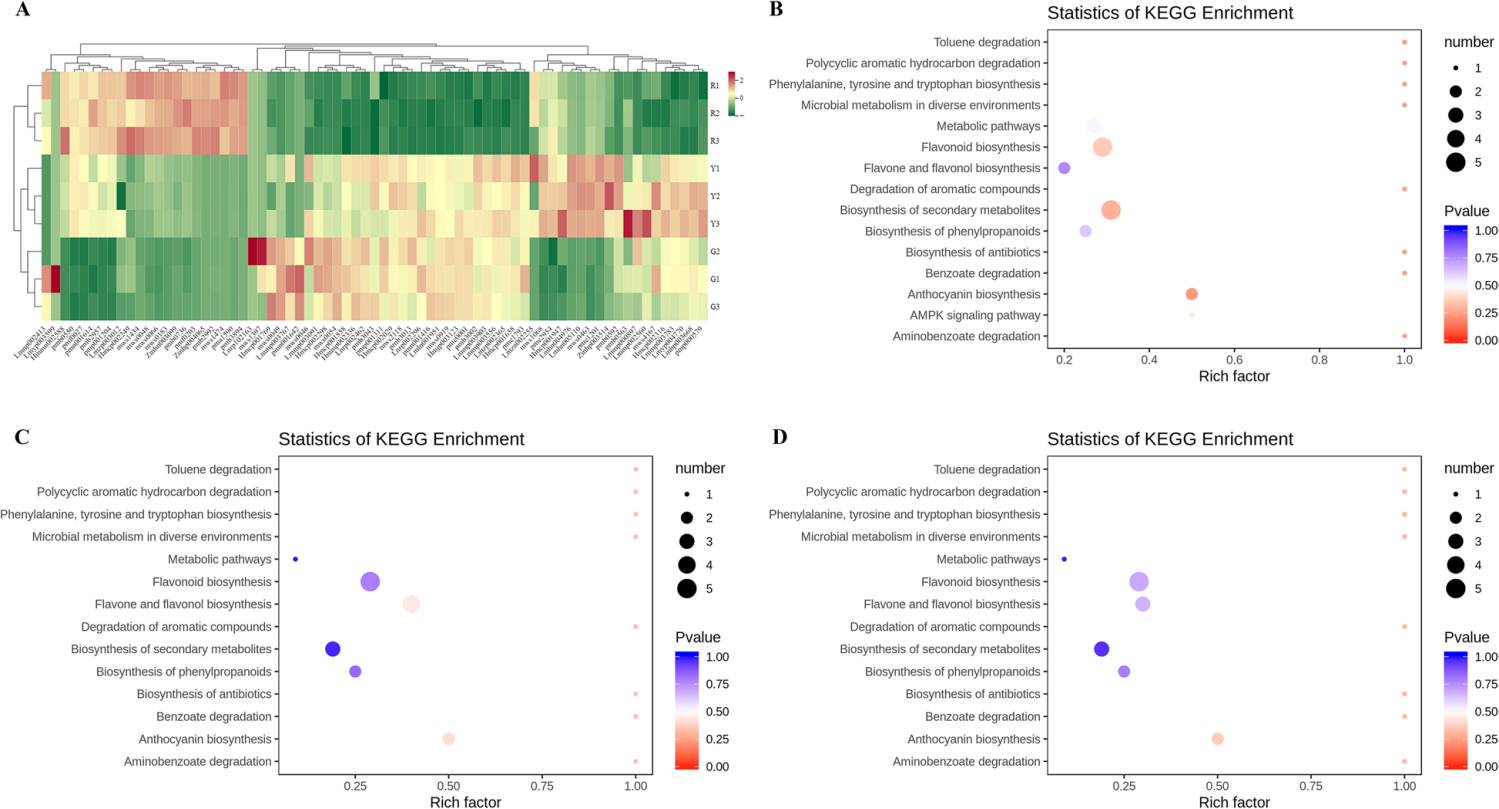
Metabolome analysis of peels at different developmental stages. **A** Heatmap of flavonoids metabolites. KEGG enrichment analysis of the DAMs between **B** G-vs-Y, **C** Y-vs-R and **D** G-vs-R

Based on UPLC-MS/MS results, the metabolomics analysis revealed significant differences in in flavonoids metabolites in peels at different developmental stages. HCA (Hierarchical cluster analysis) on the accumulation patterns of flavonoids metabolites among different samples demonstrated the good repeatability in the sample group. Heatmap analysis of flavonoids metabolites showed that 9 samples were significantly separated into three clusters corresponding to three consecutive ripening stages (green stage, yellow stage and full-red stage) (Fig. 2A). Principal component analysis (PCA) results exhibited that the G and Y stages samples were clustered together and significantly separated from the R stage samples, indicating that there were different accumulation and expression patterns of flavonoids in green, yellow and red fruits (Fig. S1). The HCA and PCA results presented that there were great differences in each treatment, and the difference between different repetitions of the same treatment was small, and the repeatability of the sample was good.

### Differentially accumulated flavonoids metabolites in ‘Hancheng Dahongpao’ peels

To investigate the differentially accumulated metabolites (DAMs) of flavonoids in the ‘Hancheng Dahongpao’ fruits at different development stages (G-vs-Y, Y-vs-R and G-vs-R), the flavonoids compounds with VIP value ≥1 and fold change ≥2 or fold change ≤0.5 were selected. Based on pair-wise comparisons of samples (G-vs-Y, Y-vs-R and G-vs-R), 27, 36 and 53 DAMs in G-vs-Y (Table S2), G-vs-R (Table S3) and Y-vs-R (Table S4) were found, respectively. Due to the interaction of flavonoids DAMs in ‘Hancheng Dahongpao’ peels, different pathways were formed, and the flavonoids DMs were annotated and assigned to the KEGG pathways (Fig. 2B-D). KEGG pathway enrichment analysis revealed that biosynthesis of secondary metabolites, phenylpropanoid biosynthesis, flavonoid biosynthesis, flavone and flavonol biosynthesis and anthocyanins biosynthesis were the main flavonoids enrichment pathways, which were enriched in these core-conserved metabolites. Therefore, we speculated that the DAMs in the above pathways might lead to flavonoids cause variations in in ‘Hancheng Dahongpao’ peels in the developmental process.

DAMs were differentially accumulated in three different groups, and the contents of 7 categories and 19 kinds of flavonoids metabolites were significantly different between green stage (G), yellow stage (Y) and full-red stage (R), including flavonol, flavone, flavanone, C-glycosylflavone, chalcones, flavanols, dihydroflavone and anthocyanins, and contained 7, 3, 1, 1, 1, 2, 2 and 2 compounds, respectively (Table 1). Among the flavonoids and dihydroflavone, all the compounds were up-regulated from G stage to Y stage. There is an increasing trend of the acacetin-o-glucuronic acid, apigenin-7,4′-dimethylether and eriodictiol-o-rhamnoside-o-pentoside-o-glucuronate throughout the development process, whereas the naringenin-7-o-triglycoside, 6,7,8-trihydroxy-5-methoxyflavone and hesperetin were decreased in the Y-vs-R peels. 6,7,8-trihydroxy-5-methoxyflavone and hesperetin demonstrated significantly higher accumulation in the yellow stage, and apigenin-7,4′-dimethylether had the highest content in the full red stage. Naringenin-7-o-triglycoside and eriodictiol-o-rhamnoside-o-pentoside-p-glucuronate in in yellow peels were 1891.15- and 5471.59-fold higher than that in the green peels, respectively. However, naringenin-7-o-triglycoside, 6,7,8-trihydroxy-5-methoxyflavone and hesperetin in full red peels were 0.46-,0.28- and 0.41- fold lower than those in yellow peels.

**Table 1:** Differentially accumulated flavonoids compounds in ‘Hancheng Dahongpao’ peels during developmental stages

Metabolite name	Class	Content			Fold Change	
		G	Y	R	G-vs-Y	Y-vs-R
Quercetin-o-rhamnoside-o-pentoside	Flavonol	1.70E+05	7.69E+04	2.79E+04	0.45	0.36
Isorhamnetin-o-Hexoside-o-malonyl-o-rhamnoside	Flavonol	1.43E+04	5.96E+04	2.53E+04	4.16	0.42
Kaempferol glucuronic acid	Flavonol	7.20E+03	1.92E+03	9.00E+00	0.267	0.01
Kaempferol	Flavonol	8.63E+04	2.92E+05	1.42E+05	3.38	0.49
Quercetin	Flavonol	2.60E+05	9.46E+05	4.50E+05	3.64	0.48
Kaempferol-3,7-o-α-L-rhamnoside	Flavonol	4.34E+04	1.00E+05	1.19E+07	2.31	118.71
Kaempferol 3-o-β-(2″-o-acetyl-β-D-glucuronide)	Flavonol	1.37E+05	3.39E+05	1.50E+04	2.48	0.04
Tetrahydroxyflavone-C-rhamnosyl-glucoside	C-glycosylflavone	5.40E+03	1.93E+04	6.59E+03	3.57	0.34
Naringenin-7-o-triglycoside	Flavanone	9.00E+00	1.70E+04	8.44E+03	1891.15	0.49
6,7,8-trihydroxy-5-methoxyflavone	Flavone	1.58E+05	4.88E+05	1.37E+05	3.08	0.28
Acacetin-o-glucuronic acid	Flavone	1.98E+03	9.20E+03	3.19E+05	4.64	34.71
Apigenin-7,4′-dimethylether	Flavone	1.49E+03	6.59E+03	5.83E+06	4.43	885.23
(+)-Gallocatechin	Flavanol	1.98E+07	7.48E+06	1.04E+06	0.38	0.14
Catechin	Flavanol	1.95E+07	9.52E+06	1.80E+06	0.49	0.19
Eriodictiol-o-rhamnoside-o-pentoside-o-glucuronate	Dihydroflavone	9.00E+00	4.92E+04	9.68E+04	5471.59	1.97
Hesperetin	Dihydroflavone	3.92E+04	1.04E+05	4.24E+04	2.65	0.41
Phloretin	Chalcone	6.24E+04	1.63E+05	6.09E+04	2.62	0.37
Peonidin o-hexoside	Anthocyanin	9.00E+00	4.06E+05	4.83E+06	45,119.63	11.89
Peonidin 3-o-glucoside	Anthocyanin	9.00E+00	4.57E+05	4.40E+06	50,739.26	9.63

The flavonols was mainly composed of kaempferol, quercetin and their glycoside derivatives, the anthocyanins were composed of peonidin o-hexoside, peonidin 3-o-glucoside, and the flavanols were consist of (+)-gallocatechin, catechin. As the fruit matured, the flavonols and flavanols were found with significantly lower, except to kaempferol-3,7-o-α-l-rhamnoside; while the content of anthocyanins showed significant increase. Anthocyanins and flavonols shared most of the biosynthetic pathway in flavonoid metabolism. In particular, dihydroquercetin was a common direct precursor for both quercetin and leucocyanidin. Peonidin o-hexoside and peonidin 3-o-glucoside was increased 45,119.63-, 50,739.26-fold in the G-vs-Y peels, and they increased 11.89- and 9.63-fold in Y-vs-R peels, respectively.

### Transcriptome analysis of peels at different developmental stages

Transcriptome sequencing was performed using the ‘Hancheng Dahongpao’ peels in three developmental stages. After raw data filtering, sequencing error rate and GC content distribution inspection, 9 samples of 64.53 Gb clean data were obtained, and the data of each sample were higher than 6 Gb. The sequencing base Q30 was more than 92.00% (Table 2). 9 sequencing libraries were constructed and named G1, G2, G3, Y1, Y2, Y3, R1, R2 and R3. PCA results of the genes expression based on the number of fragments per kilobase of exon per million fragments mapped (FPKM) values showed that all the biological replicates clustered together, indicating the high reliability of our sequencing data. The contribution rate of PC1 and PC2 was 51.99 and 39.13%, respectively (Fig. S2). Based on correlation analysis between samples, it can be observed that there were reliable biological replicates within the group between samples (Fig. S3). PCA and correlation analysis results unraveled that the samples within the group had good biological repeatability, and there were significant differences between samples.

**Table 2:** RNA sequencing data and quality control of G1, G2, G3, Y1, Y2, Y3, R1, R2 and R3

Sample	Raw Reads	Clean Reads	Clean Base(G)	Error Rate (%)	Q20(%)	Q30(%)	GC Content (%)
G1	64,031,252	63,110,950	9.47	0.02	97.76	93.28	43.36
G2	50,061,894	49,243,260	7.39	0.02	97.78	93.18	43.47
G3	55,918,654	55,020,934	8.25	0.02	97.74	93.25	43.37
Y1	52,153,394	51,356,658	7.70	0.02	97.86	93.66	43.39
Y2	51,487,354	50,542,868	7.58	0.02	97.72	93.18	43.34
Y3	51,669,070	55,320,988	8.30	0.02	97.85	93.51	43.37
R1	52,643,062	51,082,230	7.66	0.02	97.45	92.65	43.76
R2	52,400,068	51,685,472	7.75	0.02	97.43	92.61	43.71
R3	52,893,044	52,217,320	7.83	0.02	97.48	92.72	43.72

**Fig. 3:**
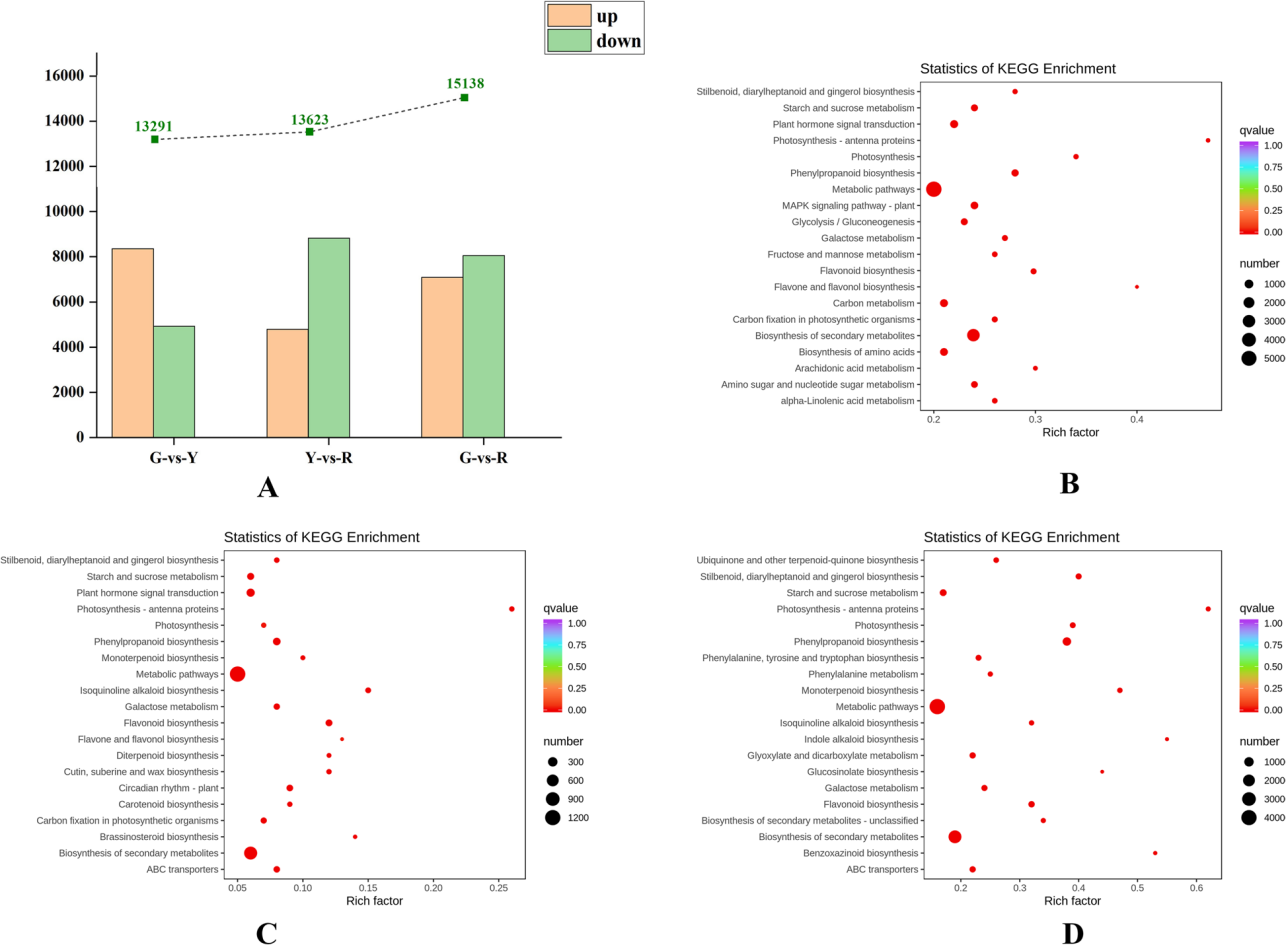
**A** Differentially expressed genes between G-vs-Y, Y-vs-R and G-vs-R. **B-D** KEGG enrichment analysis diagram of DEGs: G-vs-Y, Y-vs-R and G-vs-R, respectively

The transcriptome data of ‘Hancheng Dahongpao’ peels during the three development stages exhibit that 17,799 genes in common were expressed. There were 13,290, 13,623 and 15,140 DEGs in the comparison of G-vs-Y, Y-vs-R and G-vs-R respectively. Based on the log_2_|Fold Change| > 1 and FDR < 0.05, 8360, 4800 and 7092 genes were upregulated and 4930, 8823 and 8048 genes were downregulated in G vs Y, Y vs R, G vs R, respectively (Fig. 3A and Table S5). In order to further understand the differential genes involved in flavonoids biosynthesis, DEGs were further assigned to KEGG pathway. In KEGG analysis, DEGs mapped to metabolic pathways occupied the largest proportion, with “biosynthesis of secondary metabolites” and “plant home signal transduction” ranking second and third (Fig. 3B-D), which demonstrated that the DEGs were mainly concentrated in the secondary metabolism process. Plant home signal transduction induced the endogenous hormones variation such as ABA and brassinosteroid (BRs), and the production and accumulation of small molecule substances such as jasmonic acid (JA) and salicylic acid (SA), and converted environmental signals into endogenous signals, and made the signal transduction and amplification. Moreover, phenylalanine and small molecules induced the expression and activity of flavonoids biosynthesis pathway enzyme genes, and further promoted the flavonoids synthesis and accumulation. In detail, the pathways of the flavonoid biosynthesis and phenylpropanoid biosynthesis involved in flavonoid accumulation were apparently enriched in the mature peels. Furthermore, a quite large number of starch and sucrose metabolism encoding genes were also enriched evidently. Soluble sugar was the basic synthetic material of plant secondary metabolism, and was the direct precursor and indirect precursor of flavonoids glycosides synthesis, regulating many plant life activities through signaling pathways. In addition, the ABC transporter encoding genes are also enriched in the G-vs-Y, Y-vs-R peels, indicating that the enhanced synthesized flavonoids and needs to be transferred to a suitable organelle for storage. However, note that antenna proteins involved in photosynthesis showed the highest rich factor during all the pathways. For each group, DEGs enrichment associated with flavonoid metabolism were mapped to flavonoid biosynthesis and phenylpropanoid biosynthesis pathway, which displayed significant upregulation in the comparison of G-vs-Y, Y-vs-R and G-vs-R.

### Metabolic and gene coexpression networks in ‘Hancheng Dahongpao’ peels at different developmental stages

**Fig. 4:**
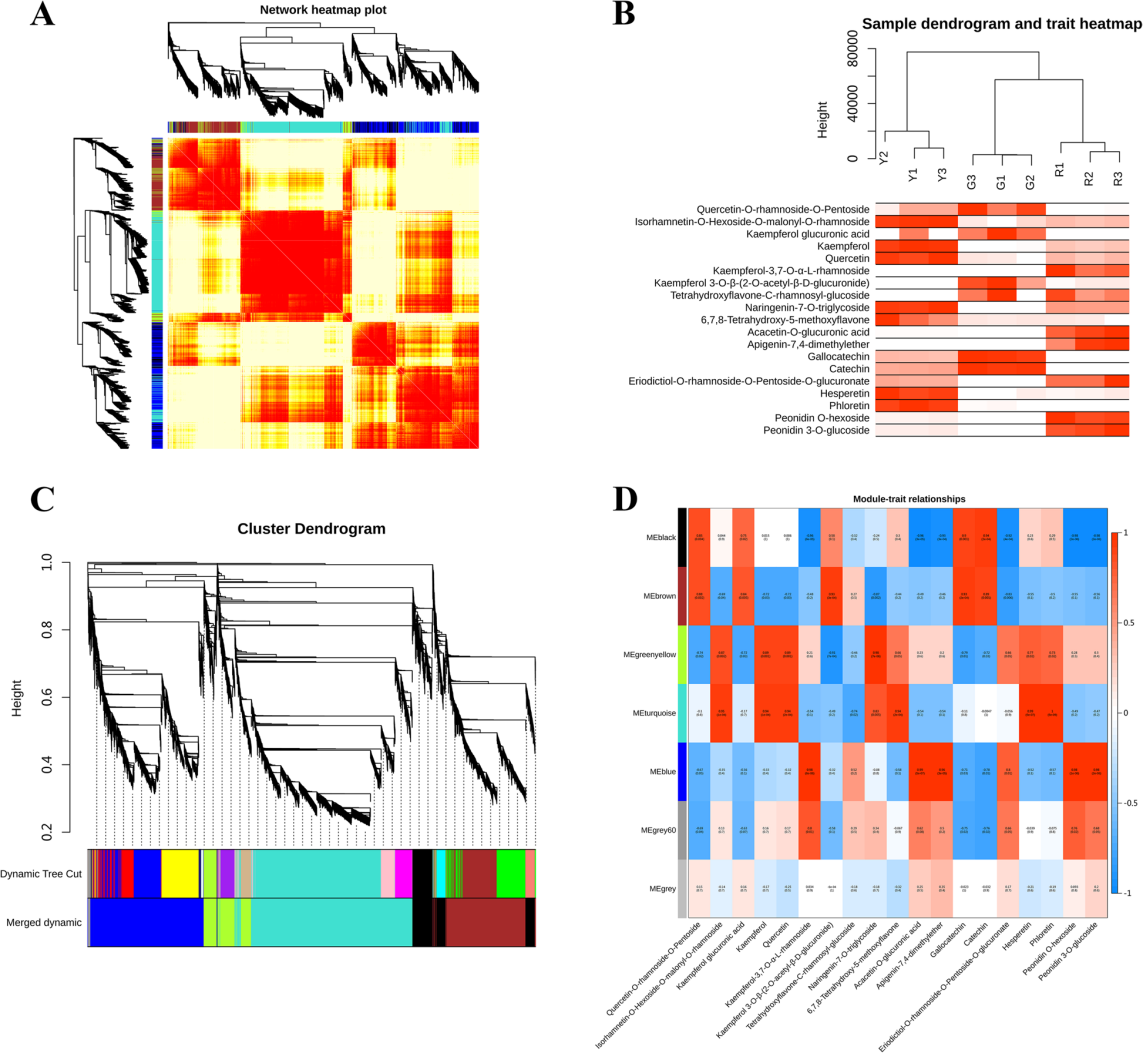
**A** Network heatmap plot of genes subjected to co-expression module calculation. **B** Heat map of 19 differential metabolites contents at three development stage. **C** Module hierarchical clustering tree based on the correlation of gene expression. Each color in the graph represents a color corresponding to each gene on the cluster tree that belongs to the same module. **D** Correlation heat map between 19 differential metabolites and gene modules. Color from blue to red indicated r^2^ values from-1 to 1

WGCNA analysis was carried out to identify co-expressed gene modules based on genes fpkm values and explore the correlation between genes in these modules and metabolites. A total of 17,799 genes were selected to use in WGCNA analysis, and the corresponding relationship between the scale independence and the mean connectivity under different thresholds were measured by the power values from 1 to 30. The scale independence and the mean connectivity of all gene adjacency functions in the corresponding gene modules showed that the best power value was 30. 7 distinct gene modules and clustering tree were built based on the correlation of gene expression (Fig. 4A). Each color in the module-level clustering tree graph represented a color corresponding to each gene on the clustering tree that belonged to the same module. The sample dendrogram and trait heatmap were constructed using the 19 differential metabolites contents at three development stage as the phenotypic data to illustrate the differential metabolites variation (Fig. 4B).

The heatmap of the correlation between gene modules and differential metabolites revealed that five gene modules were highly correlated with flavonoids. A detailed description of the correlation between 19 DAMs and gene modules was present in Fig. 4C and Fig. 4D. The blue module (4543 genes, *P* < 0.01) exhibited a strongly positive relation with peonidin o-hexoside, peonidin 3-o-glucoside, kaempferol-3,7-o-α-L-rhamnoside, acacetin-o-glucuronic acid and apigenin-7,4-dimethylether, with a correlation coefficient (r^2^) of 0.98, 0.98,0.98,0.99 and 0.96 respectively. The turquoise module (6664 genes, *P* < 0.01) was positively correlated with isorhamnetin-O-hexoside-O-malonyl-O-rhamnoside (*r*^2^ = 0.95), kaempferol (r^2^ = 0.94), quercetin (*r*^2^ = 0.94) and hesperetin (*r*^2^ = 0.99). The greenyellow module (1610 genes, *P* < 0.01) displayed significantly high correlations with quercetin (*r*^2^ = 0.89) and hesperetin (*r*^2^ = 0.89). The brown module (3239 genes, *P* < 0.01) was significantly positively related to kaempferol 3-o-β-(2-o-acetyl-β-D-glucuronide) (*r*^2^ = 0.93), gallocatechin (*r*^2^ = 0.93) and catechin (*r*^2^ = 0.89). The black module (1648 genes, *P* < 0.01) was positively linked to gallocatechin (*r*^2^ = 0.90) and catechin (*r*^2^ = 0.94).

### Differentially expressed genes linked to flavonoids biosynthesis in ‘Hancheng Dahongpao’ peels

**Fig. 5:**
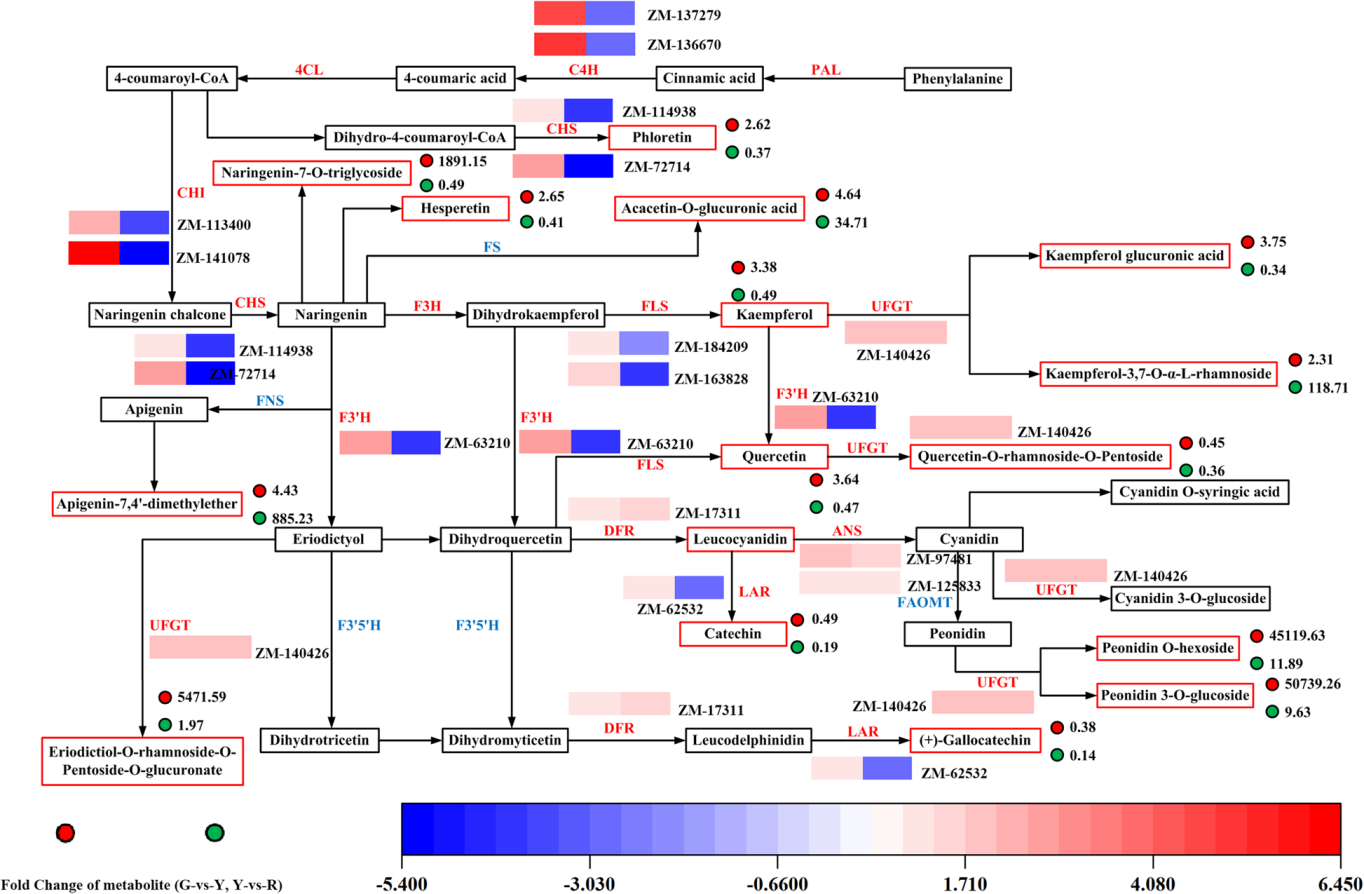
Flavonoids biosynthetic pathway in ‘Hancheng Dahongpao’ peels at different development stage. The red and green solid circles represented fold change of metabolites (G-vs-Y, Y-vs-R). Grids with color from blue to red represent log2 fold change of differentially expressed genes (G-vs-Y, Y-vs-R). PAL, phenylalanine ammonia lyase; C4H, cinnamate 4-hydroxylase; 4CL, 4-coumarate CoA ligase; CHS, chalcone synthase; CHI, chalcone isomerase; F3H, flavone 3-hydroxylase; F3’H, flavonoid 3′,-hydroxylase; F3’5’H, flavonoid 3′,5′-hydroxylase; DFR, dihydroflavonol reductase; ANS, anthocyanidin synthase; UFGT, UDP-glycose:flavonoid glycosyltransferase; FS, flavone synthase; FLS, flavonol synthase; LAR, Leucoananthcyanidin reductase

To explore the difference of flavonoid biosynthesis in ‘Hancheng Dahongpao’ peels at different developmental stages, transcriptome and metabolomics data were integrated for joint analysis (Fig. 5). Kaempferol, quercetin, kaempferol-3,7-o-α-L-rhamnoside, kaempferol 3-o-β-(2″-o-acetyl-β-D-glucuronide), naringenin-7-o-triglycoside and apigenin-7,4′-dimethylether were included in flavonoids and flavonols biosynthesis pathways. Peonidin o-hexoside and peonidin 3-o-glucoside as DAMs were involved in anthocyanin biosynthesis pathway. 14 DEGs were screened from transcriptome through enrichment analysis, and nine key structural gene families were found in phenylpropanoid biosynthesis, flavonoids and anthocyanin biosynthesis pathways, including C4H (2 DEGs), CHS (2 DEGs), CHI (2 DEGs), F3 ‘H (1 DEG), FLS (2 DEGs), ANS (2 DEGs), DFR (1 DEGs), LAR (1 DEGs) and UFGT (1 DEGs).

In the flavonoid biosynthetic pathway, the upregulation of the main trunk pathway genes and FLS (ZM-163828 and ZM-184209) in turquoise module resulted in the reinforced production of kaempferol and quercetin in G-vs-Y; the activated UDP-glycose flavonoid glycosyltransferase (UFGT) (ZM-140426) in blue module catalyzed the glycosylation of flavonol aglycones, leading to the elevated biosynthesis of kaempferol glucuronic acid, kaempferol-3,7-o-α-L-rhamnoside and quercetin-o-rhamnoside-o-pentoside. It was especially notable that flavanoid 3′-hydroxylase (F3′H) (ZM-63210) in blue module could catalyze the hydroxylation of B-ring at C3′ for various substrates, such as dihydroflavonols and flavones. Eriodictyol and apigenin were formed from narigenin catalyzed by F3′H, which could high accumulation of eriodictiol-o-rhamnoside-o-pentoside-o-glucuronate and apigenin-7,4′-dimethylether. Leucocyanidin and leucodelphinidin were catalyzed by LAR to produce catechin and (+) -gallocatechin, respectively. The upregulation of LAR (ZM-62532) gene in black module led to the the reinforced accumulation of catechin and (+)-gallocatechin. With the upregulation of CHS (ZM-114938 and ZM-72714) in turquoise module, the production of phloretin was enhanced. In the anthocyanidin biosynthesis pathway, it is notable that the structural genes of DFR (ZM-17311), LAR/ANS (ZM-125833 and ZM-97481) and UFGT (ZM-140426) in blue module were strongly up-regulated, which resulted in the significant production of various anthocyanidin glycosides finally. Peonidin o-hexoside and peonidin 3-o-glucoside were extensively generated through the biosynthetic pathway, demonstrating that two compounds were the main anthocyanins in ‘Hancheng Dahongpao’ peels resulting in the peel color change from green to full-red. However, neither increase nor evident signals were found for some important intermediates (such as leucoanthocyanidins and anthocyanidins) in the anthocyanin pathway, indicating that these metabolites might be unstable in ‘Hancheng Dahongpao’ peels.

In G-vs-Y, CHI (ZM-141078) was the most significantly upregulated DEGs in the flavonoid pathway, increasing 84.729-fold, followed by C4H (ZM-136670 and ZM-137279), which showed 32.890-fold and 23.605-fold upregulation in the yellow peels. In Y-vs-R, F3’H (ZM-158354) stood out from the DEGs with 7.655-fold change, followed by DFR (ZM-17311) and ANS (ZM-97481). In G-vs-R, the UFGT (ZM-140426) ranked first among the DEGs, increasing 13.183-fold, followed by ANS (ZM-97481) with 8.788-fold change and DFR (ZM-17311) with 6.083-fold change. These results indicated that the expression of the above structural genes was an important prerequisite for the flavonoids accumulation. In ‘Hancheng Dahongpao’ peels, flavonoids were usually converted to their glycoconjugates and then compartmentalized in vacuoles for storage, and these glycosylation reactions were catalyzed by glycosylation transferases. UDP-glycose/flavonoid glycosyltransferases (UFGT), a kind of glycosyltransferase, catalyzed the production of a wide range of substrates, such as anthocyanin and glycosides derivatives. The above results showed that the combined analysis of expression profile and metabolites constructed a clear flavonoid biosynthesis pathway during ‘Hancheng Dahongpao’ fruit development stage, and provided a strong research basis for further exploration of flavonoid metabolism in ‘Hancheng Dahongpao’ peels.

### Analysis of transcription factors in ‘Hancheng Dahongpao’ peels

The flavonoids biosynthesis in ‘Hancheng Dahongpao’ peels was not only regulated by the structural genes discussed above, but also was controlled by transcription factors. A total of 418,382 and 604 differentially expressed transcription factors were identified in G-vs-Y, Y-vs-R and G-vs-R, respectively (Table S6). The differentially expressed transcription factors were divided into various families, such as WRKY, TCP, NAC, MYB, MADs-box, HSF, HD-ZIP, GRAS, C2C2, bHLH and AP2/ERF.

**Fig. 6:**
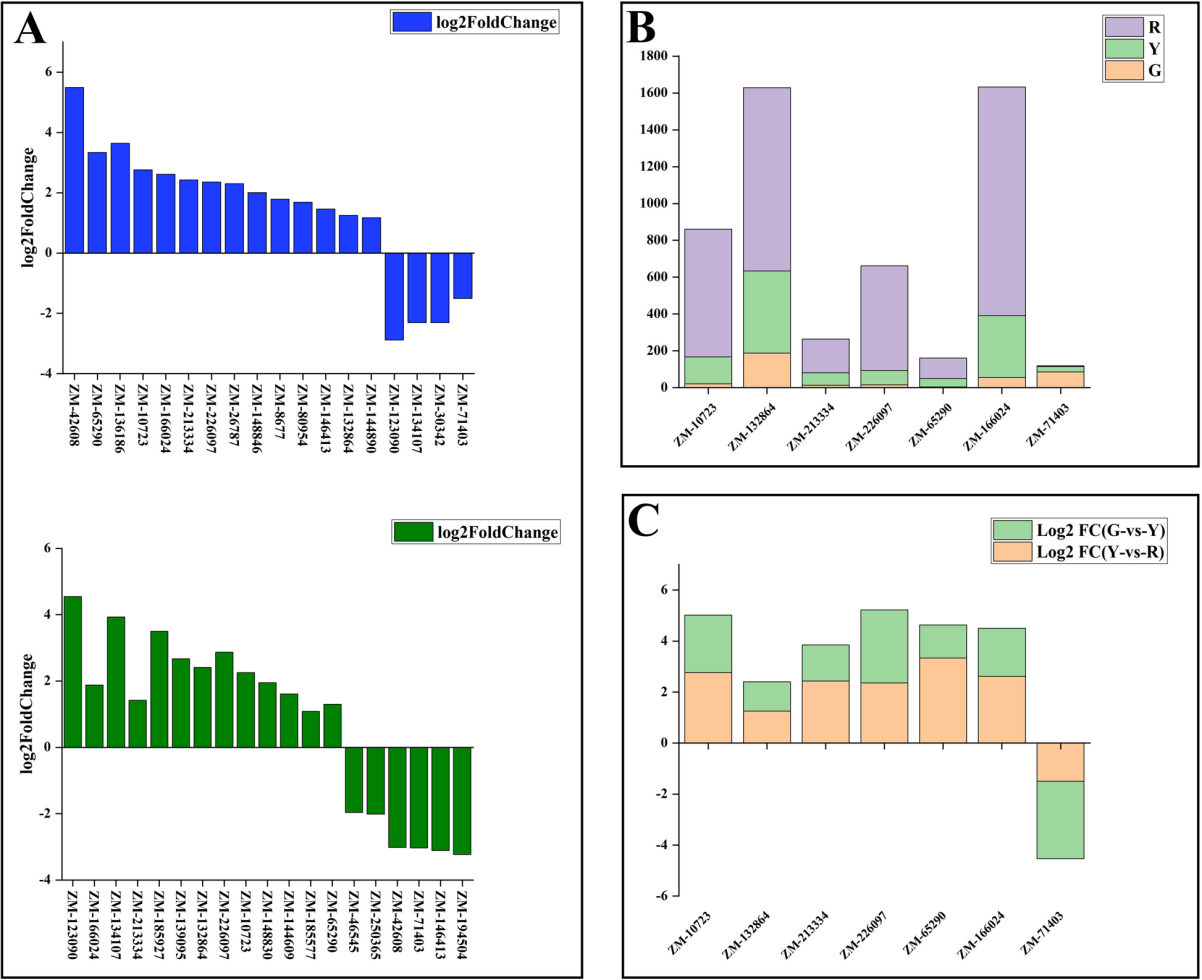
**A** Differentially MYB expressed transcription factor encoding genes between G-vs-Y (above) and Y-vs-R (below). **B** All the mutual differentially expressed MYB genes between G, Y and R. **C** Log2 FC values of the mutual MYB genes in G-vs-Y and Y-vs-R

MYB transcription factors belonged to the R_2_R_3_ MYB family, and were closely associated with flavonoids biosynthesis, which possessed obvious role in regulating flavonoids accumulation. 14 upregulated and 4 downregulated MYB transcription factors were identified in G-vs-Y, 13 upregulated and 6 downregulated MYB transcription factors were selected as significant transcription factors in Y-vs-R (Fig. 6). A sequence analysis of these MYB proteins presented that five transcription factors (MYB-ZM1, MYB-ZM3, MYB-ZM5, MYB-ZM6 and MYB-ZM7) were closely associated with flavonoids regulation **(**Fig. S4 and Fig. S5), in which the MYB-ZM5 (ZM-65290) exhibited a high sequence homology with the essential anthocyanin regulatory gene VvMYBA1, MYBA1 and MYBA2, involved in regulating anthocyanin synthesis. Multiple alignment analysis of the MYB proteins from various species showed that MYB-ZM1 (ZM-10723), MYB-ZM6 (ZM-166024), and MYB-ZM7 (ZM-71403) shared the same conserve R2 and R3 motif with the MYB transcription factors regulating the anthocyanin and flavonoids biosynthesis. MYB-ZM1and MYB-ZM7 showed a close relationship to ATMYB11, MYBF1, ATMYB12, and ATMYB111, which were involved in flavonoids biosynthesis. MYB-ZM6 exhibit a high sequence homology with VvMYBF1, which positively regulated flavonols biosynthesis. MYB-ZM3 (ZM-213334) was closely togethered with flavonoid MYB repressors, such as FaMYB1 and AtMYB4 [33]. Coincidentally, the MYB-ZM1, MYB-ZM3, MYB-ZM5, MYB-ZM6, and MYB-ZM7 were selected to be the candidate transcription factors involved in flavonoids accumulation. Altogether, these results strongly indicated that MYB-ZM1, MYB-ZM3, MYB-ZM5, MYB-ZM6 and MYB-ZM7 participated in the transcriptional regulation of anthocyanins, flavonols and flavonoids in ‘Hancheng Dahongpao’ peels.

### Correlation between DEGs and flavonoids DAMs

To further study the relationship between DEGs and DAMs in ‘Hancheng Dahongpao’ peels, the Pearson correlation coefficient between genes and metabolites were calculated. The results of correlation network showed a strong correlation (coefficients *r* > 0.8 or < −0.8) between flavonoids and their regulatory structural genes and transcription factors (Fig. S6). For example, MYB-ZM5 and the two structural genes (ANS and UFGT) were significantly positively correlated with peonidin o-hexoside and peonidin 3-o-glucoside. However, the kaempferol glucuronic acid was negatively regulated by MYB-ZM1, MYB-ZM3 and MYB-ZM6. The above results revealed that these structural genes and transcription factors played an important regulatory role in flavonoid synthesis in ‘Hancheng Dahongpao’ peels.

### RT -qPCR validation of the transcriptomic data

**Fig. 7:**
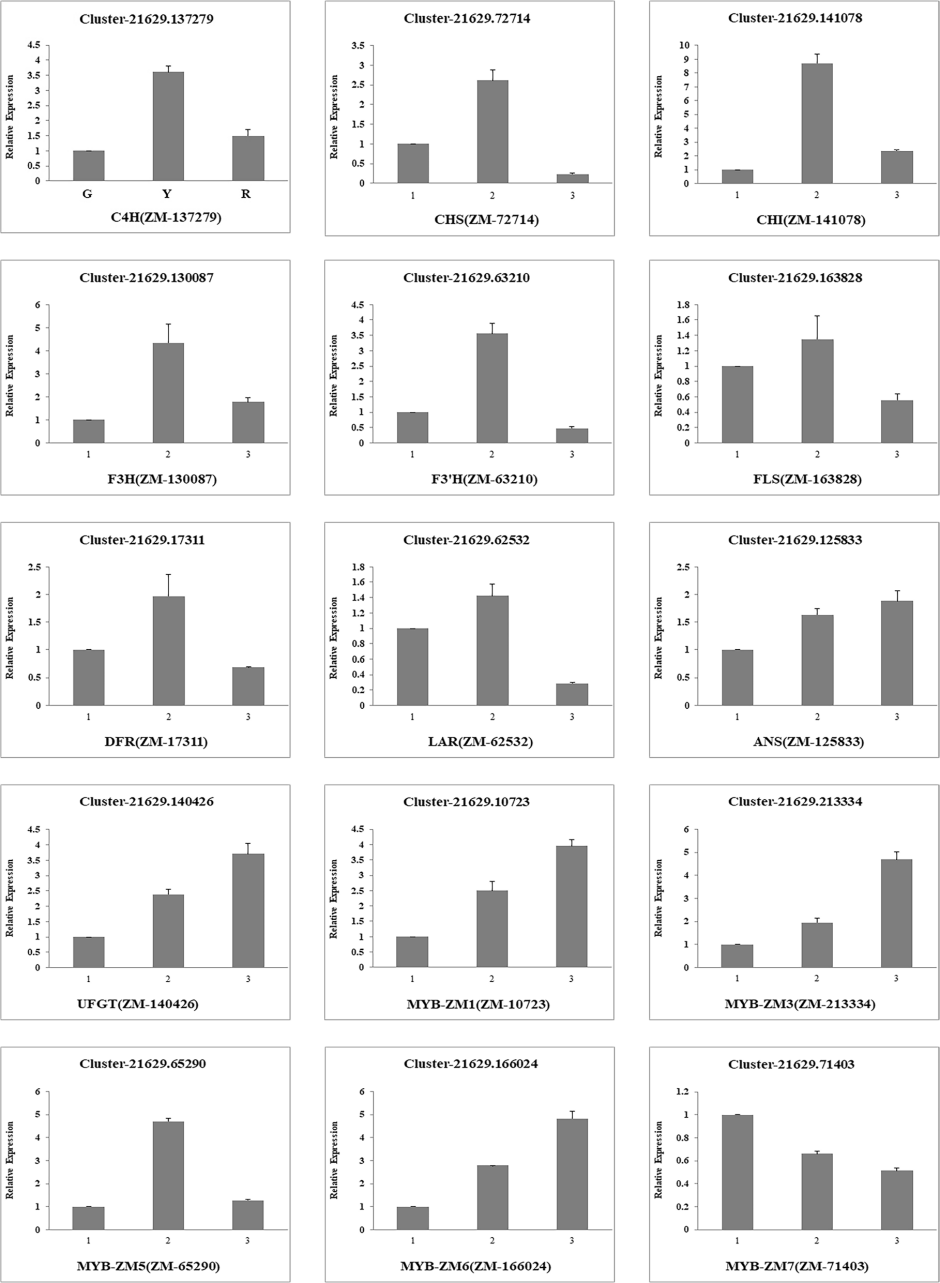
RT -qPCR verification of 10 structure differentially expressed genes and 5 MYB transcription factors expression levels. C4H (ZM-137279), CHS (ZM-72714), CHI (ZM-141078), F3H (ZM-130087), F3’H (ZM-63210), FLS (ZM-163828), DFR (ZM-17311), LAR (ZM-62532), ANS (ZM-125833), UFGT (ZM-140426), MYB-ZM1 (ZM-10723), MYB-ZM3(ZM-213334), MYB-ZM5 (ZM-65290), MYB-ZM6 (ZM-166024) and MYB-ZM7(ZM-71403) in the peels of three development stages

To further verify the candidate genes involved in flavonoids biosynthesis, 10 structure differentially expressed genes and 5 MYB transcription factors related to flavonoids biosynthesis were selected to investigate and verify their expression patterns. Consistent with the transcriptome data, both the candidate genes, including C4H (ZM-137279), CHS (ZM-72714), CHI (ZM-141078), F3H (ZM-130087), F3’H (ZM-63210), FLS (ZM-163828), DFR (ZM-17311), LAR (ZM-62532), ANS (ZM-125833), UFGT (ZM-140426), and the flavonoids regulatory genes, such as MYB-ZM1 (ZM-10723), MYB-ZM3(ZM-213334), MYB-ZM5 (ZM-65290), MYB-ZM6 (ZM-166024) and MYB-ZM7(ZM-71403), were in concordance with the transcriptome analysis, which further confirmed that these genes may be associated with flavonoids accumulation (Fig. 7 and Table S7).

## Discussion

Flavonoids have many physiological activities and are widely used in food. Chinese prickly ash has been widely concerned as a medicinal and food homologous plant, and its peels are rich in flavonoids and have great development and utilization value. The functional gene mining and molecular regulation mechanism of flavonoid biosynthesis have not been elucidated. It was expected to preliminarily reveal the related genes and metabolic pathways of flavonoids metabolism in Chinese prickly ash peel by multi-omics methods, so as to lay the foundation for the study of flavonoids in plants.

Flavonoids could be used as regulators in plant growth and development to protect plants against UV damage and prevent the invasion of pathogenic microorganisms. Isoflavones are also important phytoalexins [34]. Usually, flavonoids accumulated in Chinese prickly ash peels to protect chloroplasts from photoinhibitory and photooxidative damage by absorbing high-energy quanta [35]. In the whole development of Chinese prickly ash peel, the anthocyanins content was increased continuously, while most flavonoid differential metabolites were increased and decreased in green to yellow stage and yellow to red stage, respectively. Analysis of the differentially accumulated metabolites over the ripening stages revealed that flavonoids were the main metabolites modulating Chinese prickly ash peel reddening, and we identified several groups of flavonoids, including flavanone, flavone, flavonol, flavonoid and anthocyanins, that differentially accumulated in peels over the ripening stages. Kaempferol, quercetin, peonidin and their glycoside derivatives were the most important flavonoid compounds in Chinese prickly ash peels. In the present study, we found that peonidin o-hexoside and peonidin 3-o-glucoside were all up-regulated over the fruit ripening stages and therefore may be considered as the key anthocyanins conferring the red pigmentation of ‘Hancheng Dahongpao’ peels.

Flavonoid biosynthesis was mainly controlled by structural gene, which directly encoded various enzymes related to flavonoids secondary metabolism biosynthesis, and also was regulated by transcription factors, which were involved in regulating the expression of structural genes. Flavonoid biosynthesis were closely related to the expression of PAL, CHS, CHI, FLS, F3H, F3’H and other genes in pomegranate [36], strawberry [21], tea [37], apple [38], grape [39], asparagus [40], citrus [41] and other horticultural plants. In kiwifruit, F3H played a regulatory role in flavonoid biosynthesis by binding to transcription factors [42]. In the study of flavonoid metabolism in Ginkgo biloba, CHS, FLS, CHI, F3’H and DFR gene family, different genes of the same gene family expressed differently under dark and light conditions [43]. In this study, transcriptome data analysis showed that 26 differentially expressed genes were directly involved in flavonoid biosynthesis in ‘Hancheng Dahongpao’ peels at different development stages, The differentially expressed genes could be divided into three categories (Cluster I-III). The genes in cluster I were involved in phenylpropanoid biosynthesis, including three differentially expressed genes (C4H, CHS, CHI). Cluster II was linked to the synthesis of flavonoids and flavonols, and was composed of F3H, F3’H and FLS genes. The genes in cluster III were related to flavanols and anthocyanin synthesis, and there were four differentially expressed genes, including DFR, LAR, LDOX / ANS and UFGT. Through KEGG analysis, the core genes involved in flavonoids synthesis were C4H (ZM-137279), CHS (ZM-72714), CHI (ZM-141078), F3H (ZM-130087), F3’H (ZM-63210), FLS (ZM-163828), DFR (ZM-17311), LAR (ZM-62532), ANS (ZM-125833) and UFGT (ZM-140426). CHS, CHI and F3H in the flavonoid synthesis pathway catalyzed the upstream substances to synthesize dihydroflavonol in turn. The kaempferol and quercetin were generated under the upregulation of FLS (ZM-163828). The anthocyanin synthesis was initiated under the catalysis of F3’H and F3’5’H, respectively. During anthocyanin synthesis, DFR, LODX/ANS and UFGT played key roles in the pathways, respectively. Two ANS genes (ZM-97481 and ZM-125833) and one UFGT gene (ZM-140426) were up-regulated in the whole developmental stages, which contributed to the accumulation of peonidin o-hexoside and peonidin 3-o-glucoside. In grape, UFGT was reported to regulate anthocyanin biosynthesis during reddening, similarly as in our study [44]. The flavonoid metabolism was also controlled by transcription factors such as MYB-ZM1 (ZM-10723), MYB-ZM3(ZM-213334), MYB-ZM5 (ZM-65290), MYB-ZM6 (ZM-166024) and MYB-ZM7(ZM-71403). On the one hand, the unigenes MYB-ZM5 (ZM-65290) was clustered with VvMYBA1, MYBA1 and MYBA2, which regulated all anthocyanin biosynthesis in the various tissues of different plant species [45], and might be the candidate genes responsible for anthocyanin accumulation in Chinese prickly ash peel. On the other hand, MYB-ZM1, MYB-ZM3, MYB-ZM6 and MYB-ZM7 were closely gathered with flavonoid MYB transcription factors, such as FaMYB1 and AtMYB4, and regulated flavonoid accumulation via the transcriptional regulation of structural genes [33].

Combined metabolome and transcriptome analysis revealed the variations of flavonoids components and related regulatory genes in ‘Hancheng Dahongpao’ peels. A detailed pathway diagram of flavonoids biosynthesis of ‘Hancheng Dahongpao’ peels was constructed to better understand the relationship between genes and metabolites, and the flavonoids compounds formation. In addition, we would focus on how some changes in those gene networks regulated the biosynthesis of other nutrients and fruit economic traits in future studies.

## Conclusion

In this study, the fresh fruits of Chinese prickly ash (‘Hancheng Dahongpao’) were used as the materials to reveal the flavonoids compounds formation mechanism of ‘Hancheng Dahongpao’ fruits based on the variation pattern of flavonoids metabolites at different developmental stages. 19 differential metabolites were identified in peels. 23 differentially expressed genes identified in the 5 genes modules were highly correlated with flavonoids at different developmental stages. Through the combined analysis of differential expressed genes and differential metabolites, 15 key candidate genes involved in flavonoid biosynthesis were finally screened. Altogether, this work lay an excellent foundation for the molecular mechanism of flavonoids biosynthesis and accumulation in ‘Hancheng Dahongpao’ peels at different development stages and provided a series of candidate genes with applications in the breeding of flavonoids-rich cultivars.

## Supplementary Information

**Additional file 1.**

**Additional file 2: Table S1**. The primers and UBQ gene. **Table S2**. Differentially accumulated flavonoid compounds in the green and yellow peels. **Table S3**. Differentially accumulated flavonoid compounds in the green and full red peels. **Table S4**. Differentially accumulated flavonoid compounds in the yellow and full red peels. **Table S5**. The transcriptome data of ‘Hancheng Dahongpao’ peels and the differentially expressed genes identified in G-vs-Y, Y-vs-R and G-vs-R. **Table S6**. Differentially expressed transcription factors identified in G-vs-R. **Table S7**. RT-qPCR data of 10 structure differentially expressed genes and 5 MYB transcription factors expression levels.

## Data Availability

All data generated or analyzed during this study are included in this published article and its Supplementary information files. The data used to support the findings of this study are available from the corresponding author on reasonable request.

## Abbreviations

PAL: Phenylalanine ammonia lyase
C4H: Cinnamate 4-hydroxylase
4CL: 4-coumarate CoA ligase
CHS: Chalcone synthase
CHI: Chalcone isomerase
F3H: Flavone 3-hydroxylase
F3′H: Flavonoid 3′,-hydroxylase
F3′5′H: Flavonoid 3′,5′-hydroxylase
DFR: Dihydroflavonol reductase
ANS: Anthocyanidin synthase
UFGT: UDP-glycose:flavonoid glycosyltransferase
FS: Flavone synthase
FAOMT: Flavonoid 3′,5′-methyltransferase
3MaT1: Malonyl-CoA:anthocyanin 3-O-glucoside-6-O-malonyltransferase
FLS: Flavonol synthase
LAR: Leucoananthcyanidin reductase
UPLC-MS/MS: Ultra Performance Liquid Chromatography Tandem Mass Spectrometry.
