# Supplementary figures and images for: Regulation mechanisms of flavonoids biosynthesis of Hancheng Dahongpao peels (Zanthoxylum bungeanum Maxim) at different development stages by integrated metabolomics and transcriptomics analysis
Source: BMC Plant Biol. 2022 May 21;22:251. doi: 10.1186/s12870-022-03642-5 (PMC9123719; doi:10.1186/s12870-022-03642-5)

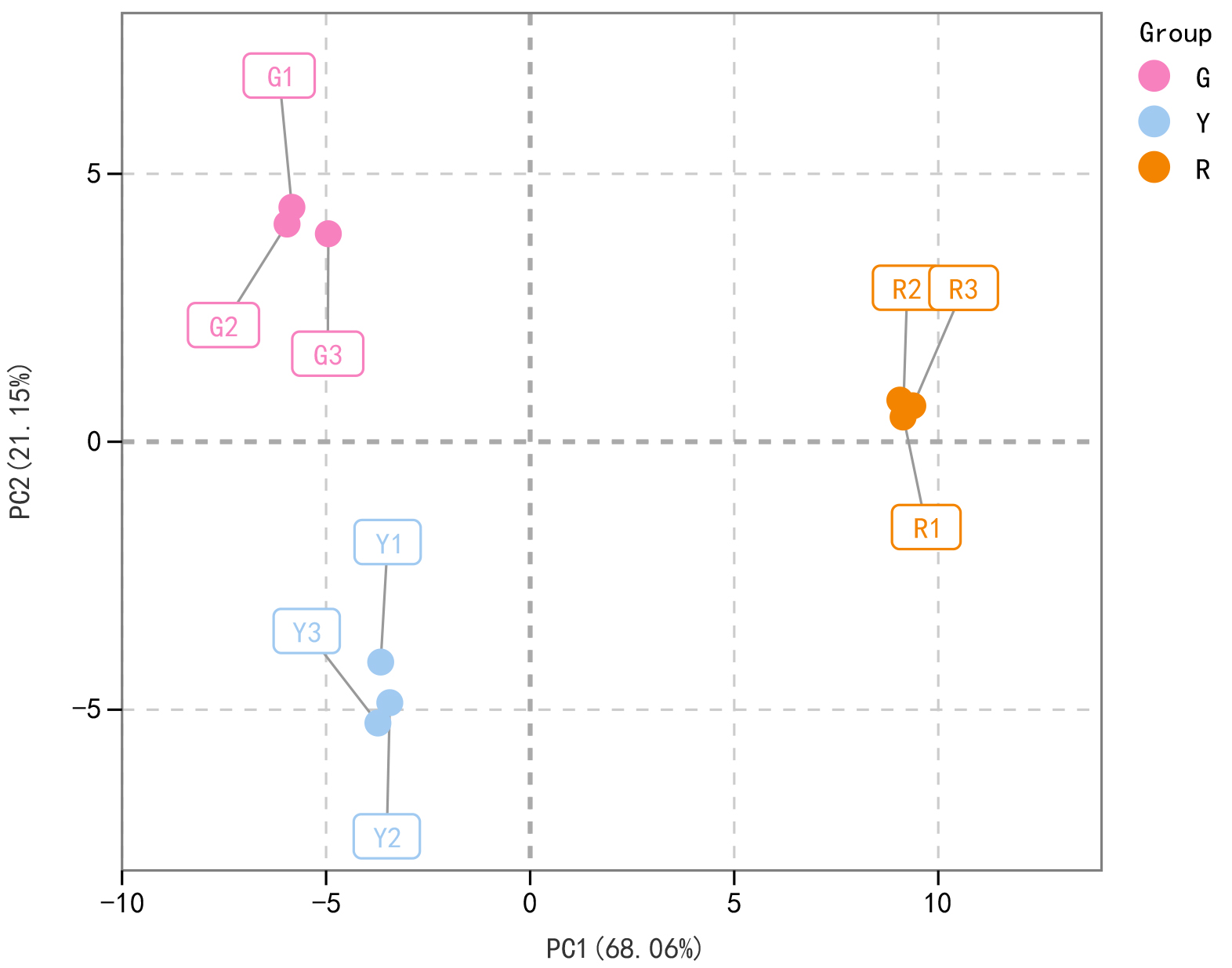

Supplement: Supplementary file 1 — Additional file 1. [file 12870_2022_3642_MOESM1_ESM.zip › Figure S1.jpg]

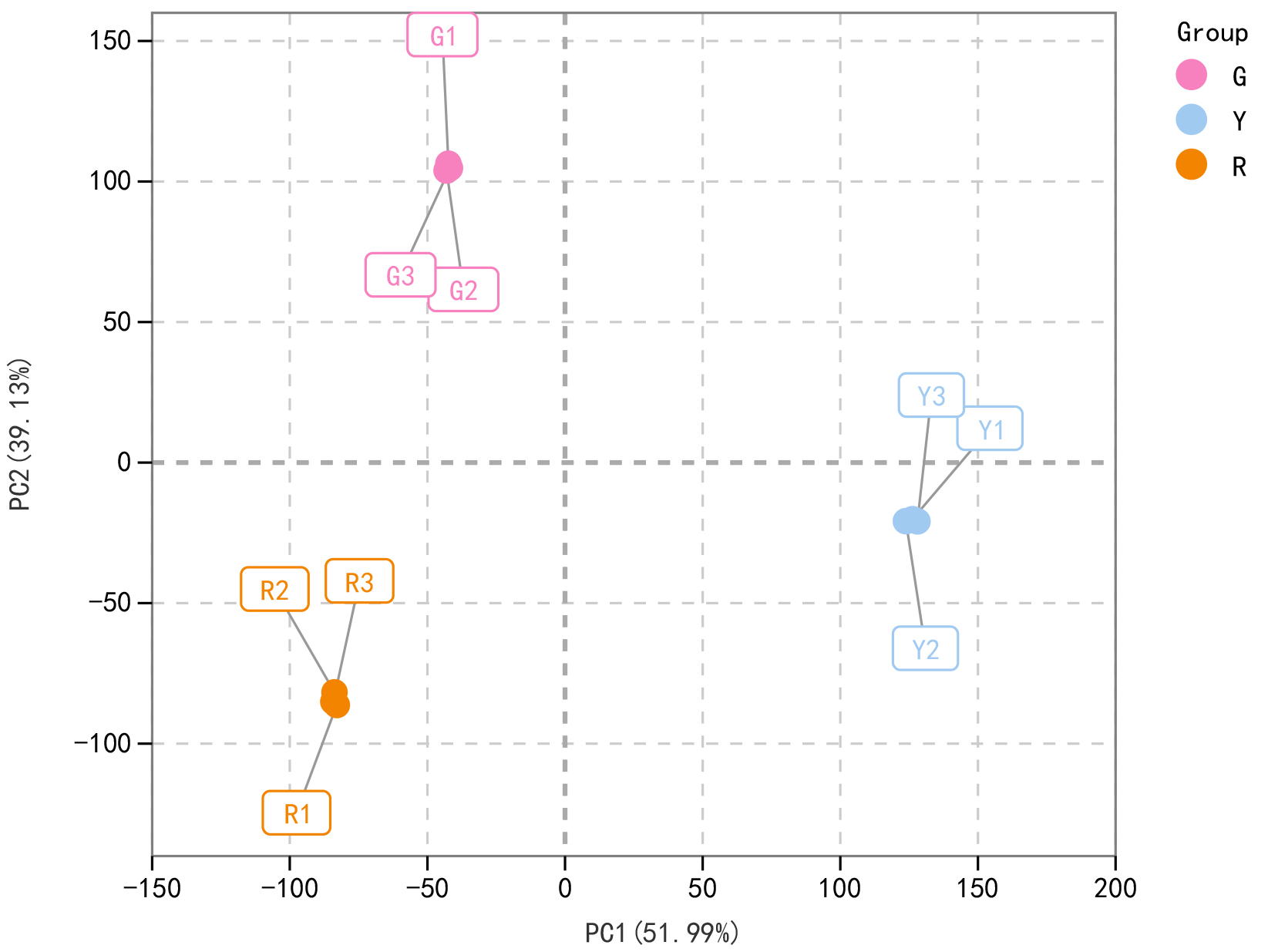

Supplement: Supplementary file 1 — Additional file 1. [file 12870_2022_3642_MOESM1_ESM.zip › Figure S2.jpg]

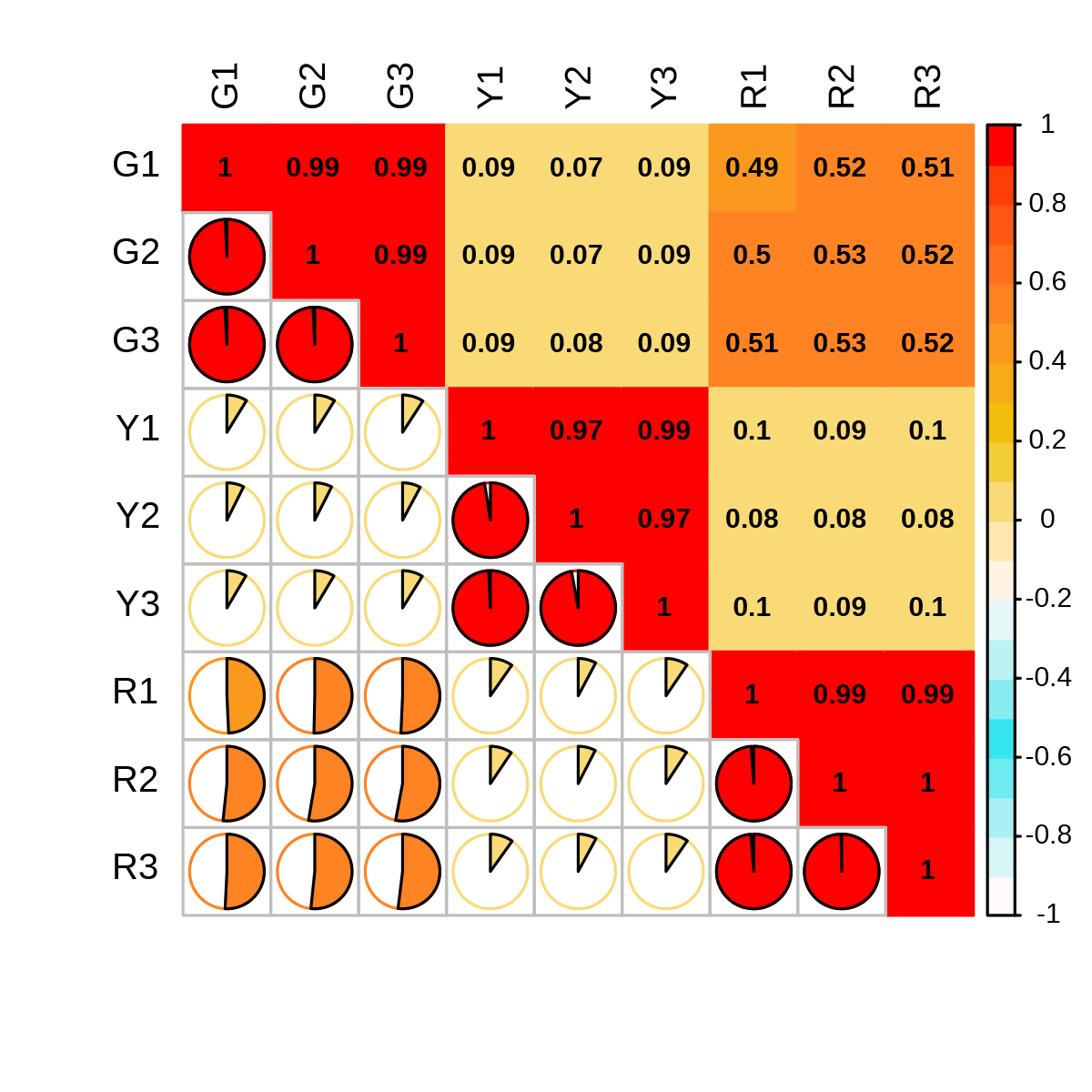

Supplement: Supplementary file 1 — Additional file 1. [file 12870_2022_3642_MOESM1_ESM.zip › Figure S3.jpg]

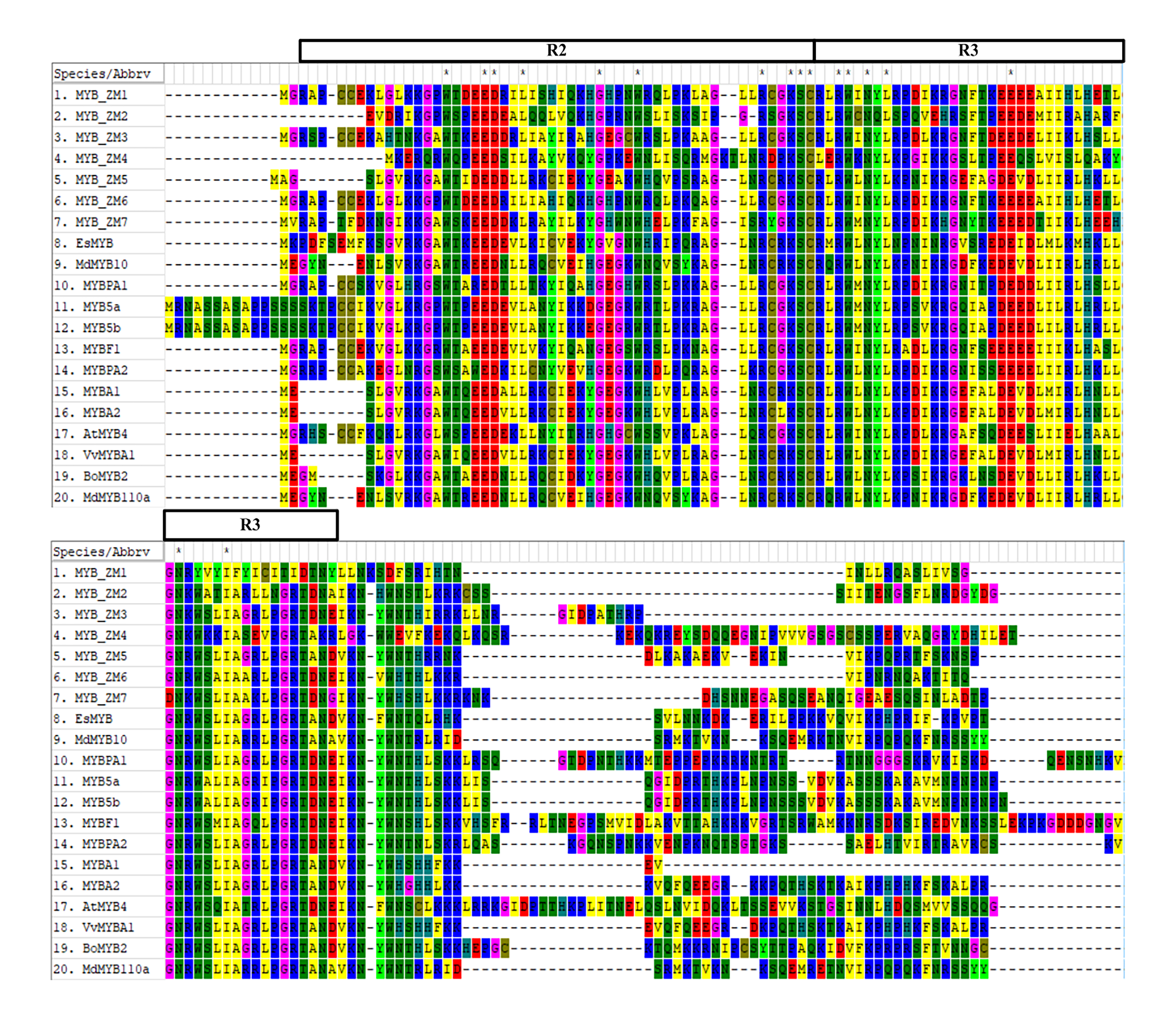

Supplement: Supplementary file 1 — Additional file 1. [file 12870_2022_3642_MOESM1_ESM.zip › Figure S4.png]

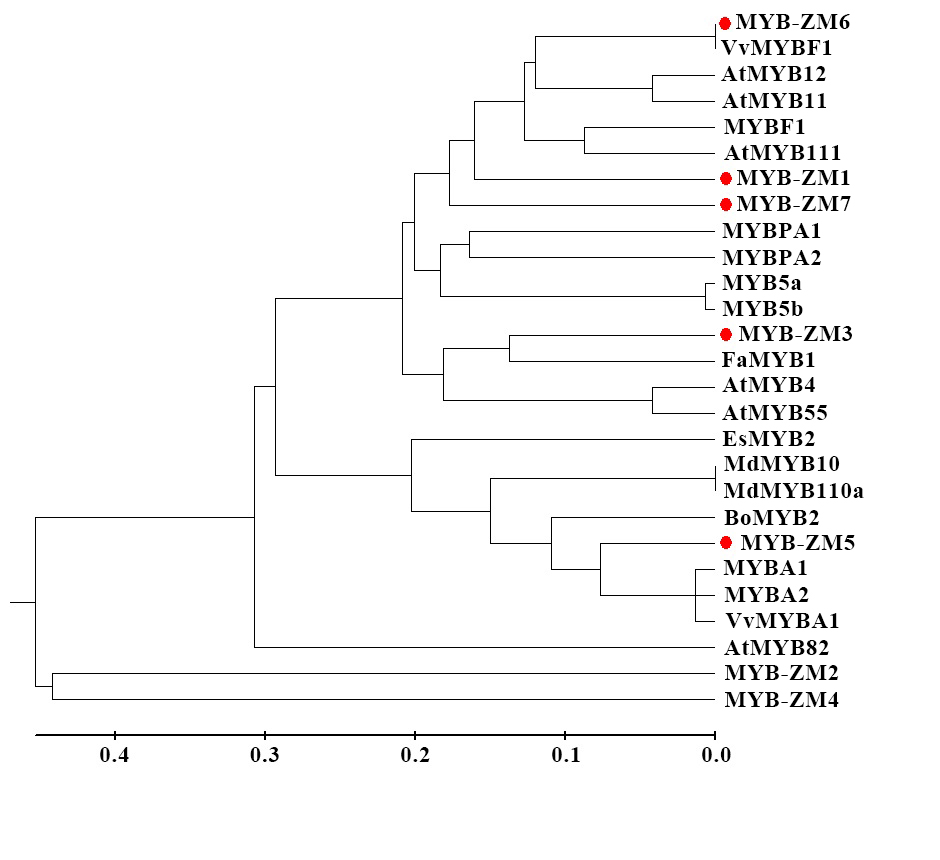

Supplement: Supplementary file 1 — Additional file 1. [file 12870_2022_3642_MOESM1_ESM.zip › Figure S5.jpg]

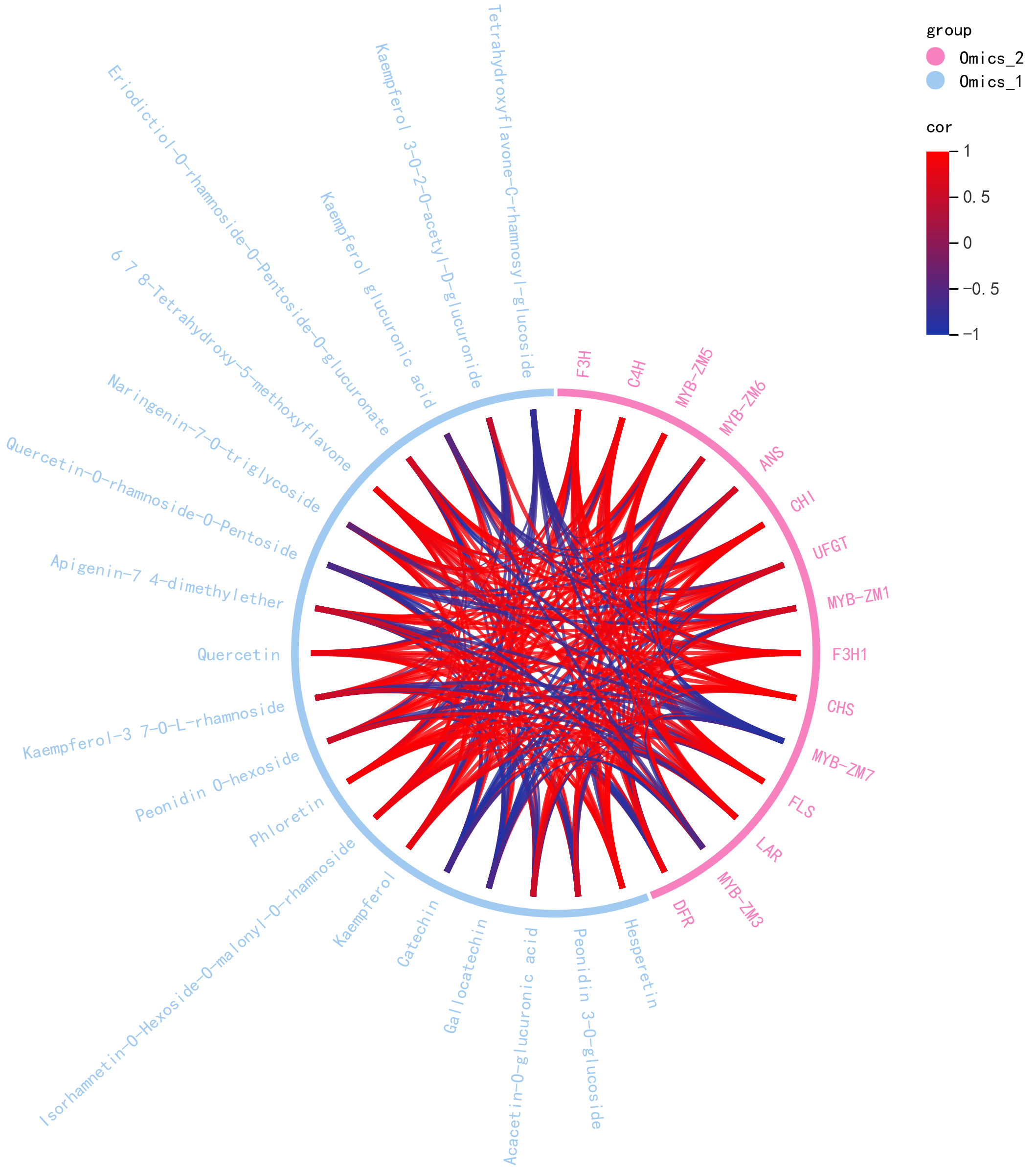

Supplement: Supplementary file 1 — Additional file 1. [file 12870_2022_3642_MOESM1_ESM.zip › Figure S6.jpg]
